# AQP4-MOG Double-Positive Neuromyelitis Optica Spectrum Disorder: Case Report with Central and Peripheral Nervous System Involvement and Review of Literature

**DOI:** 10.3390/ijms232314559

**Published:** 2022-11-23

**Authors:** Antonio Luca Spiezia, Antonio Carotenuto, Aniello Iovino, Marcello Moccia, Matteo Gastaldi, Rosa Iodice, Enrico Tedeschi, Maria Petracca, Luigi Lavorgna, Alessandro d’Ambrosio, Vincenzo Brescia Morra, Roberta Lanzillo

**Affiliations:** 1 Multiple Sclerosis Clinical Care and Research Centre, Department of Neuroscience, Reproductive Sciences and Odontostomatology, Federico II University of Naples, 80131 Naples, Italy; 2 Department of Neuroscience, Reproductive Science and Odontostomatology, Federico II University, Via Pansini, 5, 80131 Naples, Italy; 3 Neuroimmunology Laboratory, IRCCS Mondino Foundation, 27100 Pavia, Italy; 4 Department of Advanced Biomedical Sciences, Federico II University, Via Pansini 5, 80131 Naples, Italy; 5 Department of Advanced Medical and Surgical Sciences, University of Campania Luigi Vanvitelli, 80131 Naples, Italy

**Keywords:** neuromyelitis optica spectrum disorder, NMOSD, MOGAD, AQP4, radiculopathy, rituximab

## Abstract

(1) The co-occurrence of AQP4 and myelin oligodendrocyte glycoprotein (MOG) antibodies in patients with demyelinating disorders is extremely rare. In addition, a concomitant involvement of the peripheral nervous system (PNS) has been described either in association with AQP4 antibodies-positive neuromyelitis optica spectrum disorder (NMOSD), or MOG-associated disease. We report on a case of NMOSD with co-occurrence of AQP4 and MOG antibodies and concomitant central and peripheral nervous system involvement. We also reviewed available cases of AQP4-MOG double-positive patients. (2) Brain and spine MRI, cerebrospinal fluid studies, and electrophysiological test were performed. Serum AQP4 and MOG positivity was assessed with live cell-based assay. (3) A 62-year-old woman presented with recurrent optic neuritis, myelitis, and radiculitis, tested positive for AQP4 and MOG antibodies, and was treated successfully with rituximab. (4) Although few cases of AQP4-MOG double-positive patients were already described mostly affecting females with a concomitant spinal cord and optical nerve involvement, we describe the first case of double-positive NMOSD with the peculiar involvement of both central and peripheral nervous system.

## 1. Introduction

Autoantibodies against aquaporin-4 (AQP4) are the hallmark of neuromyelitis optica spectrum disorder (NMOSD), a group of immune-mediated disorders of the central nervous system (CNS) characterised by frequent involvement of the optic nerve and/or the spinal cord [1]. AQP4 antibodies have diagnostic and prognostic value. Alternatively, some patients with NMOSD phenotype can harbour antibodies against the myelin oligodendrocytic protein (MOG), that usually identifies a separate demyelinating disorder (MOG-associated disorders, MOGAD). A concomitant involvement of the peripheral nervous system (PNS) has been described in association with AQP4 antibodies [2,3,4], and, very rarely, also in patients with MOGAD. [5] In addition, the co-occurrence of AQP4 and MOG antibodies in patients with demyelinating disorders is extremely rare. Hereby, we report on a case with concomitant involvement of CNS and PNS, and the presence of both AQP4 and MOG antibodies. We also reviewed all available case reports describing clinic and laboratory features of AQP4-MOG double-positive patients.

## 2. Results

**Figure 1:**
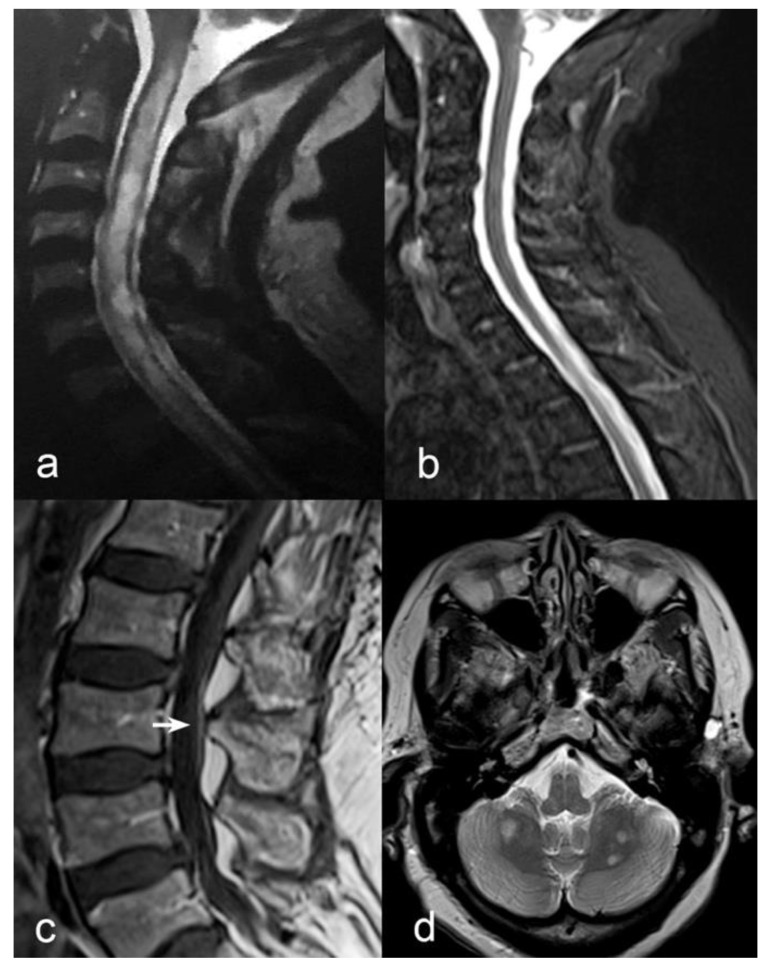
(**a**) Sagittal T2-weighted MRI of the cervical spine in the acute phase showing heterogeneous hyperintensity from C2 to the first thoracic segments with predominant central involvement. (**b**) 1-year after onset, sagittal STIR-T2-weighted MRI of cervical spine showed absence of previously seen inflammatory lesions. (**c**) Contrast enhancement of cauda equina roots (white arrow) was observed in sagittal T1-weighted sequences after Gadolinium administration. (**d**) Multiple bilateral round-shaped inflammatory lesions in cerebellar white matter were evident on axial T2-weighted axial scan (**d**).

A 62-year-old Senegalese woman initially presented with a sudden-onset reduction in visual acuity and colour perception in both eyes. In the next 4 months, in addition to the visual impairment, she developed whole-body hypoesthesia and flaccid tetraplegia with urinary incontinence. She was admitted to the Dakar University Hospital. At that time, laboratory tests showed neutrophilic leucocytosis and high C-reactive protein (167.1 mg/L). Acute infection from HIV, syphilis, hepatitis B and C viruses, cytomegalovirus, herpesvirus 1/2, Epstein–Barr virus, varicella-zoster virus, borrelia, tuberculosis, toxoplasma, rubella and measles was ruled out by serologic testing. Cerebrospinal fluid (CSF) analysis showed normal cell, glucose and protein levels. No oligoclonal bands (OCB) test was performed. Spinal cord magnetic resonance imaging (MRI) showed hyperintense T2 lesion in the central part of the spinal cord extending from C2 to T1 (Figure 1a), associated with reduced signal on T1-weighted images, without gadolinium enhancement. No brain imaging was executed. She was diagnosed with myelitis, and was commenced on azathioprine 100 mg per day, without acute phase treatment. After 1 year, she was admitted to our centre for a second opinion. Neurological examination confirmed the presence of visual impairment in both eyes (20/200 in right eye, 20/600 in left eye), and bilateral severe weakness in proximal and distal lower limbs muscles, moderate weakness of upper limbs, reduced muscle tone and areflexia at four limbs, loss of deep sensation below the clavicles, and bilateral kinetic tremor at finger-to-nose test. The patient was restricted to a wheelchair. Routine blood tests were unremarkable. CSF showed normal cell, glucose, and protein levels; isoelectric focusing revealed unique-to-CSF OCB. Serum positivity for AQP4 antibodies was titred to the endpoint (1:10,240). Serum positivity for MOG antibodies was titred to the endpoint (1:1280). Both AQP4 and MOG antibodies were negative on CSF. Whole spinal cord MRI showed regression of previously described lesions (Figure 1b) and revealed T1-gadolinium enhancement of cauda equina roots (Figure 1c) and of the anterior pial surface of conus medullaris. Brain MRI showed multiple hyperintense T2-lesion in the cerebellar white matter (Figure 1d), as well as perioptic nerve sheath T1-gadolinium enhancement.

Visual evoked potentials showed increased latency and reduced amplitude on both sides. Motor-evoked potentials showed increased central conduction time to the lower limbs. The neurographic study showed reduced motor nerve conduction velocity, normal sensory nerve conduction velocity, increased latencies of the F-wave in the lower limbs and absence of the H-reflex bilaterally, suggesting a radicular involvement. Chest computed tomography, abdominal ultrasound, antinuclear antibody, and extractable nuclear antigen were executed to exclude signs of other inflammatory CNS diseases, such as sarcoidosis or systemic lupus erythematosus. Serum tumour markers were executed and resulted in negative. Positivity for anti-Ro/SSA antibodies was found but negativity for the Schirmer test allowed us to exclude Sjogren disease. Then, treatment with rituximab was started. After 6 months from the first dose of rituximab, there was an improvement in lower limbs strength, recovery of deep tendon reflexes of lower limbs, reduction of AQP4-Ig (1:1280) and MOG-Ig (1:320) titres, significant improvement in motor central conduction time and F-wave latency, with an overall improvement in motor disability (constant bilateral assistance with canes or crutches).

## 3. Discussion

NMOSD is generally classified as an immune-mediated disease of CNS. In ≥80% of cases, NMO is caused by pathogenetic IgG autoantibodies targeting AQP4. Around 10–40% of individuals with AQP4-negative NMO show the presence of IgG autoantibodies targeting MOG. AQP4 is the most diffuse water channel in CNS that is found at the highest concentrations in perivascular and peripial astrocytic endfeet, while MOG is a CNS-specific protein expressed on the surface of oligodendrocytes [1]. Notably, the co-occurrence of MOG-IgG and AQP4-IgG is rare, and points towards distinct immunopathogeneses of these diseases.

A few cases of AQP4 and MOG double-positive patients were already described [6,7,8]. Table 1 summarizes the available cases of AQP4 and MOG double-positive, showing the clinical and radiological features of patients. Most of the patients were female (12/13), had high-grade disability, EDSS > 6 (9/13) and an extensive involvement of the spinal cord. Six patients presented with longitudinally extended transverse myelitis (LETM), two with optic neuritis (ON), and five with both LETM and ON. Most of the patients (11/13) showed brain lesions, while only two patients have normal brain scans. Beyond single case description, a large study confirmed that double-positive patients are more frequently adult females, without providing any clinical description [9]. Moreover, double-positive patients were described in Japan, showing relapsing ON with poor prognosis [10].

Similarly to previously reported cases [7,8], our case showed concomitant brain and spinal cord involvement.

Specifically, in our case, we identified radiological features typical of both AQP4-positive NMOSD (i.e., edematous longitudinally-extended lesions of the cervico-dorsal spinal cord) and MOGAD (resolution of the T2 spinal lesion, presence of multiple cerebellar white matter lesions rather than brainstem, and bilateral anterior optic nerve pathology with perineural contrast enhancement rather than posterior optic nerve involvement) [11,12,13]. Our case also showed the peculiar presence of anti-Ro/SSA antibodies. This finding was already reported by Hyun et al. [6] and Yan et al. [8] The presence of Ro/SSA antibodies is likely associated with the presence of AQP4 antibodies in NMOSD, especially in those patients presenting with clinical and laboratory features of concomitant autoimmune disorders. Furthermore, Ro/SSA positivity in NMOSD patients also correlated with disease severity [14]. Conversely, Ro/SSA positivity in MOGAD was only described in one patient diagnosed with Sjogren disease and transverse myelitis [15]. Possibly, double-positive AQP4-MOG patients display a higher propensity for autoimmunity. However, the role of Ro/SSA antibodies as a marker of disease severity in NMOSD deserves further investigation. In addition, although rarely, AQP4 positivity could be reported in paraneoplastic NMOSD with a higher propensity for multiple autoantibodies positivity [1]. In our case, we excluded paraneoplastic pathogenesis since serum tumour markers, chest CT and abdominal ultrasound were negative. However, we are aware this would not completely rule out that patient may develop tumoral lesions over the follow-up, and hence, serial imaging and serum analysis will be performed.

The major novelty of our case is the involvement of PNS, which is usually not affected in NMOSD. Both isolated AQP4-positive [2,3,4] or MOG-positive [5,16] NMOSD can be associated with radicular involvement. AQP4 is expressed in the inner part of the anterior and posterior roots and in the cauda equina adjacent to the spinal cord, while, in contrast, Schwann cells are mostly located on the periphery [2]. Moreover, human AQP4 antibodies are shown to bind antigens on the proximal part of a murine cervical radix [3], and similarly, MOG may be expressed in low quantities in the peripheral myelin and the Schwann cells in humans as has been described in rodents and primates [17]. However, it is worth mentioning that some MOGAD patients with PNS involvement showed the coexistence of serum antibodies targeting neurofascin-155, contactin-associated protein-2, or GM-1, that could underpin PNS pathology [16].

## 4. Materials and Methods

MRI of the brain and whole spine with intravenous gadolinium administration was executed. Electrophysiological tests consisted of visual-evoked potentials, motor-evoked potentials, motor and sensory nerve conduction velocity, F-wave and H-reflex assessment. Chest computed tomography, abdominal ultrasound, antinuclear antibody, and extractable nuclear antigen were executed. AQP4 and MOG antibodies were assessed in serum and CSF. Serum positivity for AQP4 antibodies was assessed using an in-house live cell-based assay (CBA) transfected with an M23 AQP4 construct. Positivity was confirmed using a commercial fixed CBA at 1:10 dilution (Euroimmun). Serum positivity for MOG antibodies was assessed using an in-house live CBA transfected with a full-length EGFP-tagged MOG construct. In addition, positivity was confirmed using an IgG1 secondary antibody to ensure specificity [18].

## 5. Conclusions

NMOSD can present with double positivity to AQP4 and MOG antibodies, and with mixed clinical features, including optic neuritis, longitudinally extensive transverse myelitis, and radicular involvement. Collection and presentation of double-positive AQP4 and MOG NMOSD cases can shed light on the immunopathogenesis of this condition. However, it is unclear if MOG-AQP4 double-positive patients could represent a distinct clinical phenotype within NMOSD.

## Figures and Tables

**Table 1:** Review of available case series.

	Sex	Age	Onset Phenotype	N° Relapses	Current EDSS	Other Antibody	Spine Lesion	Brain Lesion	Treatment
Hyun et al., 2017 [6]	M	32	LETM	4	7	Anti-SS-A Ab (+), antinuclear Ab (+), speckled pattern	NA	None	AZA
Höftberger et al., 2014 [7]	F	58	LETM + ON	1	2	No	C1-C5	None	RTX
	F	50	LETM + ON	1	2	No	C4-T2	Aspecific WML	AZA
Yan et al., 2016 [8]	F	15	LETM + ON	3	7	No	C4-conus	Large MS-like lesions	CS, AZA
	F	36	LETM	8	8	No	T1-conus	Small MS-like lesions	RTX
	F	18	LETM + ON	8	8.5	Anti-SS-A A/B, Anti-Tg, anti-TPO	C1-conus	Small MS-like lesions	Met HCQ
	F	60	LETM	6	8.5	No	C2-conus	Small MS-like lesions	CS, AZA
	F	49	LETM	3	7.5	No	C1-conus	Small MS-like lesions	CS, AZA
	F	33	ON	6	3	No	C3-conus	Large MS-like lesions	CS, AZA
	F	15	ON	7	1	No	C1-conus	ADEM-like	CS, AZA
	F	20	LETM + ON	10	6.5	No	C1-conus	ADEM-like	CS, AZA
	F	30	LETM	4	7.5	No	C1-conus	ADEM-like	RTX
	F	36	LETM	6	8	No	C1-T12	Small MS-like lesions	RTX
Ishikawa et al., 2019 [10]	F	24	ON	3	NA	NA	NA	NA	CS, Iv Ig, AZA

Ab, antibody; ADEM, acute disseminated encephalomyelitis; AZA, azathioprine; CS, corticosteroids; EDSS, Expanded Disability Status Scale; F, female; HCQ, hydroxychloroquine; Iv Ig, Intravenous immunoglobulins; JC, juxtacortical white matter; LETM, longitudinally extensive transverse myelitis; M (gender), male; Met, Methotrexate; MS, multiple sclerosis; NA, not available; NMOSD, neuromyelitis optica spectrum disorder; ON: optic neuritis; RTX, rituximab; tg, thyroglobulin; TPO, thyroperoxidase; WML, white matter lesion.

## Data Availability

Not applicable.
